# The serological diversity of serum IgG/IgA/IgM against SARS‐CoV‐2 nucleoprotein, spike, and receptor‐binding domain and neutralizing antibodies in patients with COVID‐19 in Japan

**DOI:** 10.1002/hsr2.572

**Published:** 2022-04-13

**Authors:** Yudai Kaneko, Akira Sugiyama, Toshiya Tanaka, Kazushige Fukui, Akashi Taguchi, Kenji Tatsuno, Aya Nakayama, Kazumasa Koga, Yoshiro Kishi, Wang Daming, Chungen Qian, Fuzhen Xia, Fan He, Liang Zheng, Yi Yu, Youichiro Wada, Yoshiaki Wada, Tatsuhiko Kodama, Takeshi Kawamura

**Affiliations:** ^1^ Research Center for Advanced Science and Technology (RCAST) The University of Tokyo Tokyo Japan; ^2^ Medical & Biological Laboratories Co. Ltd Tokyo Japan; ^3^ Isotope Science Center The University of Tokyo Tokyo Japan; ^4^ Department of Neurology Nissan Tamagawa Hospital Tokyo Japan; ^5^ Suzhou Institute of Biomedical Engineering and Technology Chinese Academy of Sciences Suzhou China; ^6^ Department of Biomedical Engineering, The Key Laboratory for Biomedical Photonics of MOE at Wuhan National Laboratory for Optoelectronics—Hubei Bioinformatics & Molecular Imaging Key Laboratory, Systems Biology Theme College of Life Science and Technology, Huazhong University of Science and Technology Hubei China; ^7^ Reagent R&D Center Shenzhen YHLO Biotech Co. Ltd Guangdong China

**Keywords:** antibody, COVID‐19, neutralizing antibody, patient, SARS‐CoV‐2

## Abstract

**Background:**

We compared the temporal changes of immunoglobulin M (IgM), IgG, and IgA antibodies against severe acute respiratory syndrome coronavirus 2 (SARS‐CoV‐2) nucleoprotein (N), spike 1 subunit (S1), and receptor‐binding domain (RBD), and neutralizing antibodies (NAbs) against SARS‐CoV‐2 in patients with coronavirus disease 2019 (COVID‐19) to understand the humoral immunity in COVID‐19 patients for developing drugs and vaccines for COVID‐19.

**Methods:**

A total of five confirmed COVID‐19 cases in Nissan Tamagawa Hospital in early August 2020 were recruited in this study. Using a fully automated chemiluminescence immunoassay analyzer, we measured the levels of IgG, IgA, and IgM against SARS‐CoV‐2 N, S1, and RBD and NAbs against SARS‐CoV‐2 in COVID‐19 patients' sera acquired multiple times in individuals from 0 to 76 days after symptom onset.

**Results:**

IgG levels against SARS‐CoV‐2 structural proteins increased over time in all cases but IgM and IgA levels against SARS‐CoV‐2 showed different increasing trends among individuals in the early stage. In particular, we observed IgA increasing before IgG and IgM in some cases. The NAb levels were more than cut‐off value in 4/5 COVID‐19 patients some of whose antibodies against RBD did not exceed the cut‐off value in the early stage. Furthermore, NAb levels against SARS‐CoV‐2 increased and kept above cut‐off value more than around 70 days after symptom onset in all cases.

**Conclusion:**

Our findings indicate COVID‐19 patients should be examined for IgG, IgA, and IgM against SARS‐CoV‐2 structural proteins and NAbs against SARS‐CoV‐2 to analyze the diversity of patients' immune mechanisms.

## 1 INTRODUCTION

To understand the immune response against severe acute respiratory syndrome coronavirus 2 (SARS‐CoV‐2) infections, measurements, and monitoring of antibodies (mainly immunoglobulin g [IgG] and IgM) against SARS‐CoV‐2 have already been performed, and some studies reported that antibodies are useful diagnostic tools for SARS‐CoV‐2 infections.^1^, ^2^ However, the chronological measurements of antibody isotypes against SARS‐CoV‐2 structural proteins and NAbs against SARS‐CoV‐2 in Japanese individuals have not been performed. Therefore, in this study, we measured temporal changes in the IgG, IgA, and IgM antibodies against SARS‐CoV‐2 N, S1, and receptor‐binding domain (RBD) and NAbs against SARS‐CoV‐2.

A total of five men in their 20–50 s with COVID‐19 confirmed in Nissan Tamagawa Hospital (Table 1), Tokyo, Japan, in August 2020 were enrolled in this study. At least seven serum samples for each patient were collected from 0 to 76 days after symptom onset. Levels of IgG, IgA, and IgM against SARS‐CoV‐2 N, S1, and RBD and NAb against SARS‐CoV‐2 were measured using a fully automatic CLIA analyzer, iFlash3000 (kits and an analyzer from Shenzhen YHLO Biotech Co.). The cut‐off value for indicating a positive test result as used by the manufacturer for all kits was 10 AU/ml.

**Table 1:** Symptoms exhibited by the patients in this study.

Symptoms											
No.	Age range	Sex	Ct value	Fever	Cough	Sore throat	Dysgeusia	Headache	Diarrhea	Dyspnea	
P1	20–29	Male	32.9	Yes	Yes	Yes	No	No	Yes	No	
P2	40–49	Male	26.6	Yes	No	No	No	Yes	No	Yes	
P3	30–39	Male	22.3	No	No	No	Yes	No	No	No	
P4	50–59	Male	18.6	Yes	No	Yes	No	No	No	Yes	
P5	30–39	Male	30.5	Yes	No	No	No	No	No	No	

**Figure 1:**
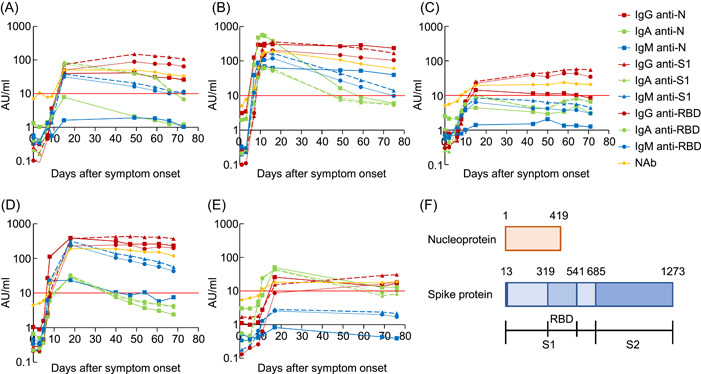
Timeline of IgG (dotted line)/IgA (dashed line)/IgM (solid line) antibody levels against N (square)/S1 (triangle)/RBD (circle) antigens of SARS‐CoV‐2 and NAb (diamond) against SARS‐CoV‐2 after symptom onset in P1 (A), P2 (B), P3 (C), P4 (D) and P5 (E). Thick lines show the cut‐off value (10 AU/ml) indicating a positive result for all kits. (F) Schematic representation of the SARS‐CoV‐2 Spike protein domain. AU, arbitrary units; N, nucleoprotein; NAb, neutralizing antibody; RBD, receptor‐binding domain; S1, spike 1 subunit; S2, spike 2 subunit; SARS‐CoV‐2, severe acute respiratory syndrome coronavirus 2

IgG levels against SARS‐CoV‐2 increased after symptom onset in all patients with COVID‐19 but levels of IgM and IgA against N and S1 exhibited different increasing trends among patients (Figure 1A–F). For example, in Patient 5, IgM and IgG levels were low but IgA levels were high on Day 11. A recent study reported that IgA levels in serum increased soon after symptom onset with mild symptoms while that a case with the severity of symptoms showed a delayed but very strong IgA response against SARS‐CoV‐2.^3^ Furthermore, measurement of serum IgA, besides IgM and IgG, improved diagnostic accuracy for SARS‐CoV‐2 infections.^4^ We also observed higher NAb levels in patients with severe symptoms than NAbs in patients with the mild symptoms. The NAb levels were reported to increase after SARS‐CoV‐2 infection in most individuals^5^ and be associated with clinical disease severity,^6^, ^7^ confirming the results of our studies. IgA and NAb levels against SARS‐CoV‐2 could be biomarker for COVID‐19 severity.

The NAb kit has the advantages of detecting angiotensin‐converting enzyme 2 (ACE2) competitively binding to RBD‐coated particles with all antibodies having neutralization activity against SARS‐CoV‐2 structural proteins while a typical antibody kit against components detects each isotype, not reflecting total NAb levels. In fact, we observed NAb levels keeping above the cut‐off value more than around 70 days after symptom onset in all COVID‐19 patients some of whose antibody levels against RBD were lower than the cut‐off value (Patient 2, Patient 3, and Patient 5). Furthermore, some of the antibody isotypes against RBD fall below 10 AU/ml in early stage, while NAb levels exceed the cut‐off value in 4/5 patients. For example, in Patient 3, the levels of any antibody isotypes against RBD were less than 10 AU/ml when NAb levels over the cut‐off value from Day 9 to Day 11 after symptom onset. The NAb kit could be as useful as IgG, IgA, and IgM kit against S1 and RBD for the accurate measurement of antibodies having neutralizing activity in patients' sera with COVID‐19.

However, there are some limitations in this study. First, we detected NAbs against only RBD of SARS‐CoV‐2 in serum samples. Antibodies against the S1 region except for RBD of SARS‐CoV‐2 were reported to have neutralizing activity.^8^, ^9^ We should detect NAbs by using S1‐coated particles, including the RBD region for analyzing NAbs. Second, we found the persistence of NAbs in patients with COVID‐19 around 70 days after symptom onset but we have not measured the neutralization activity of antibodies for a longer time yet. Some longitudinal studies have reported that neutralization activity against SARS‐CoV‐2 significantly declined as early as 6 weeks and that persisted as late as 5 months after symptom onset.^10^, ^11^ Furthermore, the binding surface in SARS‐CoV‐2 RBD to ACE2 is reported to be less antigenic than that of other S regions^12^ so the antibody levels against RBD could go down earlier than antibody levels against S1, which may affect chronological changes in NAb levels. Further longitudinal analysis of COVID‐19 patients is needed to understand immune memory after SARS‐CoV‐2 infection and vaccine.

Our results demonstrated the serological diversity of serum IgG, IgA, and IgM antibodies against SARS‐CoV‐2 N, S1, and RBD and NAbs against SARS‐CoV‐2 in patients with COVID‐19 and the necessity of combinational measurements of the antibody isotype levels against each structural protein of SARS‐CoV‐2 and NAb levels to further elucidate the immune mechanism of COVID‐19.

## AUTHOR CONTRIBUTIONS

*Conceptualization*: Tatsuhiko Kodama and Takeshi Kawamura. *Data curation*: Yudai Kaneko, Kenji Tatsuno, and Takeshi Kawamura. *Formal analysis*: Yudai Kaneko and Kenji Tatsuno. *Funding acquisition*: Youichiro Wada, Tatsuhiko Kodama, and Takeshi Kawamura. *Investigation*: Yudai Kaneko, Kazushige Fukui, Akashi Taguchi, Aya Nakayama, Akira Sugiyama, and Toshiya Tanaka. *Methodology*: Wang Daming, Chungen Qian, Fuzhen Xia, Fan He, Liang Zheng, and Yi Yu. *Project administration*: Akira Sugiyama, Toshiya Tanaka, Kazumasa Koga, Youichiro Wada, Yoshiaki Wada, Tatsuhiko Kodama, and Takeshi Kawamura. *Resources*: Kazumasa Koga. *Supervision*: Tatsuhiko Kodama and Takeshi Kawamura. *Visualization*: Yudai Kaneko.

## CONFLICTS OF INTEREST

Yudai Kaneko and Yoshiro Kishi are employed by Medical & Biological Laboratories Co. Ltd., a company that imported the testing material used in this study from Shenzhen YHLO Biotech Co. Ltd. Fuzhen Xia, Fan He, Liang Zheng, and Yi Yu belong to Shenzhen YHLO Biotech Co. Ltd., a manufacturer of diagnostic reagents used in this study. 2019‐nCoV IgM and IgG chemiluminescence immunoassay (CLIA) kits, Covid‐2019 IgG/IgA/IgM Kit against N/S1/RBD antigens, and iFlash‐2019‐ nCoV NAb were sponsored by Medical & Biological Laboratories Co. Ltd. and Shenzhen YHLO Biotech Co. Ltd. based on the joint research contract between Medical & Biological Laboratories Co. Ltd. and Shenzhen YHLO Biotech Co. Ltd., and Medical & Biological Laboratories Co. Ltd. and the University of Tokyo, respectively.

## ETHICS STATEMENT

This study was performed at the University of Tokyo and Nissan Tamagawa Hospital approved by their ethics committee (protocol number R2‐05 and Tama2020‐003), and informed consent was obtained from all participants individually.

## TRANSPARENCY STATEMENT

The corresponding author, Takeshi Kawamura confirms that manuscript is an honest, accurate, and transparent account of the study being reported.

## Data Availability

Data available on request from the authors.

## References

[1] Infantino M , Grossi V , Lari B , et al. Diagnostic accuracy of an automated CLIA for anti‐SARS‐CoV‐2 IgM and IgG antibodies: an Italian experience. J Med Virol. 2020;92(9):1671‐1675. 10.1002/jmv.25932 32330291 PMC7264663

[2] Jin Y , Wang M , Zuo Z , et al. Diagnostic value and dynamic variance of serum antibody in coronavirus disease. Int J Infect Dis IJID Off Publ Int Soc Infect Dis. 2019;94:49‐52. 10.1016/j.ijid.2020.03.065 PMC7194885 32251798

[3] Dahlke C , Heidepriem J , Kobbe R , et al. Distinct early IgA profile may determine severity of COVID‐19 symptoms: an immunological case series. medRxiv. 2020. 10.1101/2020.04.14.20059733

[4] Ma H , Zeng W , He H , et al. Serum IgA, IgM, and IgG responses in COVID‐19. Cell Mol Immunol. 2020;17(7):773–775. 10.1101/2020.04.17.20064907 32467617 PMC7331804

[5] Lau EHY , Tsang OTY , Hui DSC , et al. Neutralizing antibody titres in SARS‐CoV‐2 infections. Nat Commun. 2021;12(1):63. 10.1038/s41467-020-20247-4 33397909 PMC7782739

[6] Jeewandara C , Jayathilaka D , Gomes L , et al. SARS‐CoV‐2 neutralizing antibodies in patients with varying severity of acute COVID‐19 illness. Sci Rep. 2021;11(1):2062. 10.1038/s41598-021-81629-2 33479465 PMC7819970

[7] Legros V , Denolly S , Vogrig M , et al. A longitudinal study of SARS‐CoV‐2‐infected patients reveals a high correlation between neutralizing antibodies and COVID‐19 severity. Cell Mol Immunol. 2021;1‐10:318‐327. 10.1038/s41423-020-00588-2 PMC7786875 33408342

[8] Wan J , Xing S , Ding L , et al. Human‐IgG‐neutralizing monoclonal antibodies block the SARS‐CoV‐2 infection. Cell Rep. 2020;32(3):107918. 10.1016/j.celrep.2020.107918 32668215 PMC7332464

[9] Chi X , Yan R , Zhang J , et al. A neutralizing human antibody binds to the N‐terminal domain of the Spike protein of SARS‐CoV‐2. Science. 2020;369(6504):650‐655. 10.1126/science.abc6952 32571838 PMC7319273

[10] Prévost J , Gasser R , Beaudoin‐Bussières G , et al. Cross‐sectional evaluation of humoral responses against SARS‐CoV‐2 spike. Cell Rep Med. 2020;1(7):100126. 10.1016/j.xcrm.2020.100126 33015650 PMC7524645

[11] Wajnberg A , Amanat F , Firpo A , et al. Robust neutralizing antibodies to SARS‐CoV‐2 infection persist for months. Science. 2020;370(6521):1227‐1230. 10.1126/science.abd7728 33115920 PMC7810037

[12] Hattori T , Koide A , Noval MG , et al. The ACE2‐binding interface of SARS‐CoV‐2 spike inherently deflects immune recognition. J Mol Biol. 2021;433(3):166748–166758. 10.1101/2020.11.03.365270 33310017 PMC7833242

